# The East London Hospital for Children

**Published:** 1893-04-29

**Authors:** 


### THE EAST LONDON HOSPITAL FOR CHILDREN.

This institution was founded upwards of a quarter of a century ago in Ratcliff Cross by a young medical man, Dr. Heckford, owing to his having experienced the miserable inefficiency of the provision in that locality for nursing sick children, which the cholera epidemic in 1866 made painfully apparent. Dr. Heckford died on the day that the committee decided to purchase the site upon which the present hospital stands. It is remarkable that so young a man should display such self-denying zeal in the cause of young children, and his early decease conveys a lesson and a warning to all who would do something during their lifetime to leave the world a little better than they found it. Charles Dickens visited the hospital in December, 1868, and wrote an account of what he saw, under the title of "A Small Star in the East." We remember perfectly well visiting the old hospital in Ratcliff Cross many years ago, and the quaint character of the building. The original buildings were nothing but an old sale-loft, and our recollection of the ingenuity which was displayed in converting this loft into a hospital ward is still with us. Everything connected with the original building, which ultimately contained forty beds, impressed the visitor with the urgency of the need, which emboldened the devoted founders of this institution to attempt to treat disease under conditions so unfavourable, although, bad as they were, they proved infinitely better than the heap of straw which often constituted the only bed of the suffering children in the neighbouring alleys. The first patient received was indeed taken from the kennel of a house situated in Gin Alley. Charles Dickens urged his readers to go and see the work, as it was then, for themselves. We would earnestly urge our readers to go and see the work of the East London Hospital, Shadwell, and the North-Eastern Hospital, Shoreditch, as it is now, for themselves also. No words can drive home the pathos which the visitor encounters in the wards of these two institutions. Every cot is full. Some cots contain more than one child. Many of the children are grievously afflicted, but all of them display the ameliorating effects of the devoted service which the medical staff, and especially the sisters and nurses, render to their little charges. If there is one work which appeals more directly to the sympathies of healthy men and women than another, it is surely the work of succouring little children and alleviating their sufferings. The two hospitals for children in East London cry aloud to Londoners of every district for help and sympathy. Will that cry be allowed to continue without adequate results, as it has, alas! been allowed to continue since the days of Charles Dickens even until the present day? We do most earnestly appeal to every one of our readers who is in sympathy with children—and which of them is not?—to make it their business to pay an early visit to both of these institutions, and then to interest themselves, and to interest their friends in the work they are doing so that it may be extended, and that its efficiency may be increased to the utmost without a moment's unnecessary delay.

### General Administration.

It would not be fair for us to carefully inspect various hospitals, as we have been doing during the last few years, without drawing attention to points in their administration which strike us as affording room for improvement. There was abundant evidence of genuine kindness and sympathy for the children throughout the East London Hospital. During the time we were there a few of the children were being carried through the corridors to the house-surgeon's room, in order to attend a little tea-party, which he had kindly arranged for their amusement and benefit. This kindly act spoke eloquently of the genuine interest which was taken in the welfare and comfort of the patients. Still, a careful inspection of the hospital impressed us with the feeling that there was a slackness in various directions which might usefully be removed. It is perhaps a little difficult to state in words the precise grounds upon which this conclusion is based. Matters, trivial in themselves, when repeated in ward after ward, or the adjacent offices, reveal to the trained administrator an absence of strict supervision, and of insistence upon the best work, which none but those who have been long engaged in the administration of hospitals can adequately appreciate. So we make bold to say, that although there was nothing seriously wrong, the atmosphere of the place conveyed the impression that those responsible for the general supervision of the wards lacked thoroughness. We hope we may frankly state our impression without offence, that it may be taken in good part, and that it may lead to an improvement in certain directions, which our experience leads us to feel would prove wholly desirable. We were struck in passing up the staircase to the dormitories occupied by the nurses with an odour which led us to examine the pipes and sanitary arrangements somewhat closely. Our impression is that they require attention, and that the soil-pipes especially need modification, so as to permit a free current of fresh air to pass through them continuously.

### The Wards.

There are three wards, the largest of which contains upwards of thirty cots. One of them is devoted to boys, another to girls, and the third to infants from birth. This last constitutes a special feature of the hospital, as no other hospital in England contains such a ward, and so the nurses here gain an experience in the nursing of sick infants which may be regarded as unique. The wards have thorough cross-ventilation, and are pleasant and attractive. No doubt wards constructed like those at the North-Eastern Hospital are necessarily somewhat cold, and so require an abundance of artificial heat. This is supplied by central fireplaces, which help to brighten the ward, and supply an abundance of heat. We thought the atmosphere of the wards was somewhat high at the time of our visit, but this may have been due to accidental causes. There were an abundance of beautiful flowers, attractive pictures, and a few musical boxes. All these were contributed by the nurses or their friends. This is an eloquent fact, which reveals once more the devotion shown by the majority of women who give up their lives to nursing, and at the same time sadly reflects upon the governors, and especially upon the women governors, who have heretofore failed to relieve the nurses of a pleasant duty, which unfortunately entails a considerable expenditure out of their too limited incomes. We direct the attention of the Committee of Management to the fact, that without the nurses, these wards would often be without pictures, without flowers, without a musical box. Is it not desirable to establish some system which shall cause all these necessaries to be supplied independently of the nurses, as an encouragement to them in the faithful discharge of their arduous duties? We were further struck by the absence of toys at this hospital. At Sheffield, Sunderland, Northampton, Bradford, and throughout the provincial hospitals toys abound. Each hospital has a toy-room, and the toys are changed every day, so that the children's recovery is often considerably expedited by the cheerfulness and amusement which the little sufferers derive from the skilful use of the contents of the toy-room. May we hope that a few good people will combine together and agree to furnish a toy-room for the East London Hospital for Children, so that many a weary hour may be wiled away pleasantly, which otherwise could not fail to drag and discourage, and so perchance to delay recovery?

### Special Cots.

A visit to a children's hospital often offers many subjects of interest. We always make a point of noticing to what extent the Sunday and other schools in the surrounding district are encouraged to take an interest in the sick children by establishing cots in the various wards. We noticed that although there were several memorial cots, no Sunday or other school has, so far, displayed a personal interest in the little sufferers in this hospital. At Northampton the children's ward has been furnished with everything by the Sunday and other schools of the town and county. Surely here is a small gold mine which the authorities might work with great advantage to the hospital, and no little benefit to the children in the schools of East London. We are glad to find, however, that some children have interested themselves in the "Princess Mary" and "Enfield" wards, each of which contains a "Little Folks" cot, both of which were endowed by the readers of this magazine, who collectively subscribed no less a sum than £1,892 for this purpose. Other children may have contributed, judging by the fact that there is a "Shepperton" cot in the Enfield ward, and a "Blackheath" cot in the Enfield, and also in the "Princess Mary" ward; whilst some of the wards contain a "Canadian" cot, a "Chiswick" cot, a "Finchley" cot, a "Newbury," a "Richmond," a "Shortlands," a "Streatham," and an "Upper Tooting" cot. It may interest our readers to state that the readers of *Little Folks* made up their splendid gift out of the sixpences and shillings, which they refrained from spending on dolls and sweets to enable them to provide a refuge during sickness for little children less fortunate than themselves. Any one can endow a cot in perpetuity by a gift of £1,000, and for the lifetime of the donor for a gift of £300. Any one may endow a cot for one year by a subscription of £50. Bearing in mind the large number of schools of all kinds to be met with in the district which the East London Hospital especially serves, we hope that before long very many more cots will be endowed by Sunday and other schools at this institution.

